# Spontaneous regression of cutaneous squamous cell carcinoma and in-transit metastases following cessation of ruxolitinib

**DOI:** 10.1016/j.jdcr.2024.01.016

**Published:** 2024-01-28

**Authors:** Luke Paterson, Benjamin Paterson, Vishak Surendra, Christopher Powell

**Affiliations:** a Te Whatu Ora Health, Te Tai Tokerau, Whangārei, New Zealand; b Wellington Blood and Cancer Centre, New Zealand

**Keywords:** cutaneous squamous cell carcinoma, immunosuppression, spontaneous regression, ruxolitinib

## Introduction

Ruxolitinib is a Janus kinase (JAK) inhibitor used in the treatment of myeloproliferative neoplasms and autoimmune dermatological conditions.^1^ Ruxolitinib increases the risk of developing squamous cell carcinoma (SCC) and has been implicated in aggressive SCCs.^2^, ^3^, ^4^ Several mechanisms for ruxolitinib inducing SCC have been suggested, including impairment of immunosurveillance.^4^, ^5^, ^6^ We describe a ruxolitinib exposed patient with aggressive, recurrent SCC who had complete clinical and histological spontaneous regression following cessation of ruxolitinib.

## Case Report

A 77-year-old man was diagnosed with postpolycythemia vera myelofibrosis (MF) and started on ruxolitinib 20 mg oral twice daily, in November 2020. He has Fitzpatrick skin type II and no history of previous SCC. He was diagnosed with SCC of his scalp in November 2022. The SCC was clinically 25 mm wide and histologically poorly differentiated with a Breslow thickness of at least 8 mm on initial biopsy.

Ten weeks after initial diagnosis, he proceeded to a wide local excision of the SCC with en bloc resection of the periosteum which was covered with a transposition flap. A subcutaneous nodule was found during the raising of the flap and biopsied. Histology from this excision showed poorly differentiated SCC with a Breslow thickness of 9 mm and perineural invasion. The deep margin was clear but there were positive radial margins. The biopsied nodule confirmed in-transit metastatic subcutaneous SCC. Seventeen weeks after initial diagnosis, he represented with an ulcer within the flap corresponding to the initially biopsied in-transit metastasis. He had developed 9 additional painful, biopsy confirmed subcutaneous in-transit metastases within the scalp. A staging computed tomography (CT) at this time showed multiple in-transit metastases with no significant calvarial invasion and no distant metastatic disease. Ruxolitinib was ceased 19 weeks after initial diagnosis.

**Fig 1:**
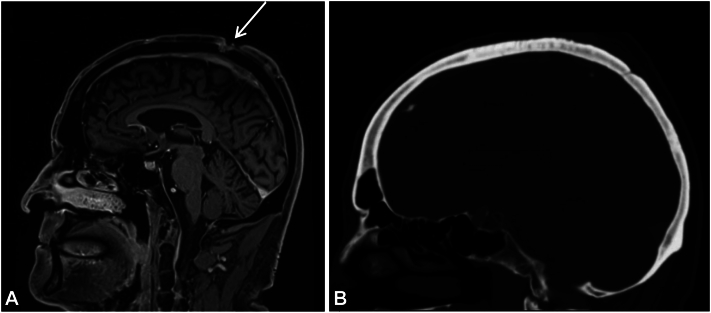
**A,** Magnetic resonance imaging head (T1, sagittal). *Arrow* indicates area of outer calvarial erosion of skull vertex. **B,** Computed tomography head (sagittal) 7 weeks earlier without evidence of calvarial erosion.

The patient met with the radiation oncology, medical oncology and surgical teams to discuss management options, and decided to proceed with surgical management of his scalp in-transit metastases. Whilst the patient was consulting the various teams, he noted subjective improvement in terms of the pain, size, and number of the multiple in-transit metastases. These findings were also noted on objective clinical examination. The patient had an magnetic resonance imaging head 24 weeks after initial diagnosis, which demonstrated areas of subcutaneous enhancement within the superior frontal scalp corresponding to in-transit metastases (Fig. 1, *A*). The number and volume of these had decreased significantly compared to the CT scan 7 weeks earlier (Fig. 1, *B*). This indicated that radiologically the in-transit disease was regressing. Somewhat contradictorily the magnetic resonance imaging demonstrated a new full thickness calvarial lesion. This may be the result of the magnetic resonance imaging being more sensitive than CT at assessing calvarial erosion or the result of disease progression on ruxolitinib, which was not ceased until 2 weeks after the CT.

**Fig 2:**
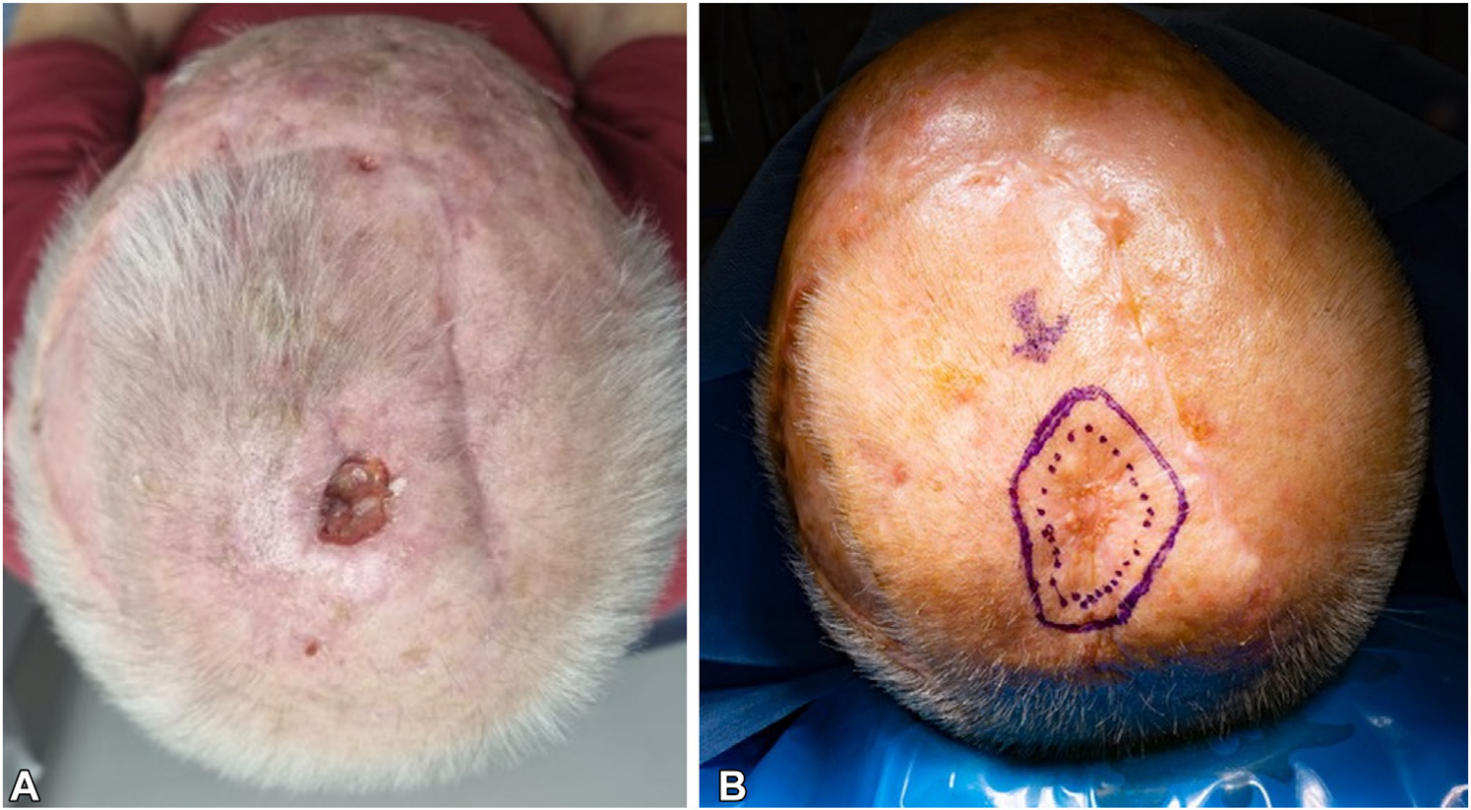
**A,** Squamous cell carcinoma recurrence with ulcer over vertex 2 weeks following cessation of ruxolitinib. **B,** Preoperative image of healed vertex ulcer 9 weeks following cessation of ruxolitinib.

**Fig 3:**
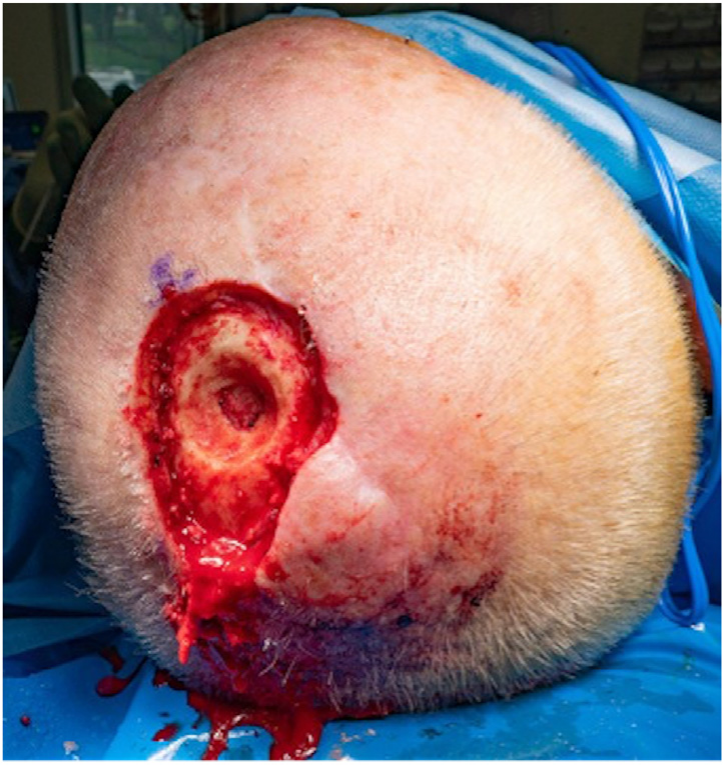
Intraoperative photo of resected ulcer and calvaria.

The patient presented for surgery 28 weeks after initial diagnosis and 9 weeks following the cessation of ruxolitinib. On the day of surgery, the in-transit metastases had clinically regressed and were no longer palpable. The scalp ulcer had completely healed (Fig. 2, *B*). Wide local excision of the healed ulcer, calvarial resection, and dural biopsy were performed (Fig. 3). The defect was covered with a transposition flap. Histological analysis of the resected (previously ulcerated) nodule, inner table of calvarium, and dural margin all returned negative for malignancy with only scar tissue present. From these findings we conclude that the SCC and in-transit metastases had clinically, histologically, and radiologically completely (and spontaneously) regressed following cessation of the ruxolitinib.

**Fig 4:**
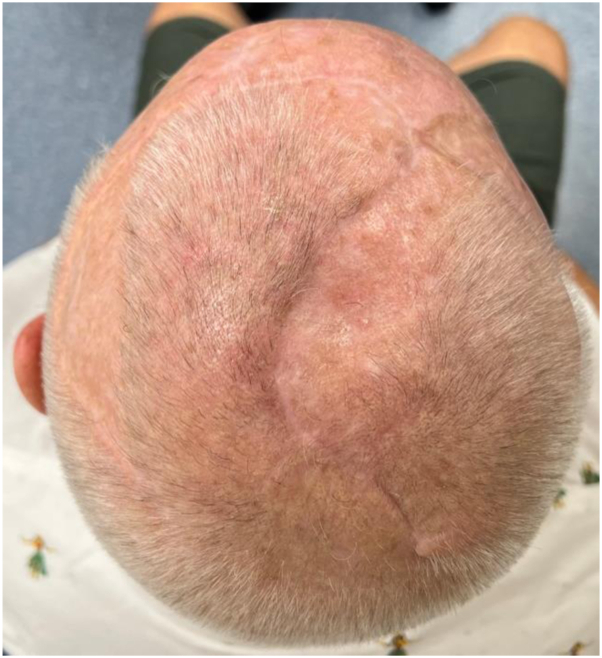
Scalp 29 weeks after surgery, no evidence of clinical recurrence.

An magnetic resonance imaging head, performed 41 weeks after initial diagnosis, showed no evidence of recurrence. At clinic follow-up, 57 weeks after initial diagnosis, there was no evidence of clinical recurrence (Fig. 4).

## Discussion

Ruxolitinib is a JAK inhibitor and was the first to be approved for use in MF. It has been shown to improve quality of life, reduce splenomegaly and improve survival in patients with MF.^1^ However, ruxolitinib does increase the risk of developing nonmelanoma skin cancers.^2^ A recent 10-year retrospective cohort study showed that for patients with polycythemia vera or MF, those exposed to ruxolitinib were more likely to develop SCC (hazard ratio of 3.24). Additionally, ruxolitinib has been implicated in the development aggressive SCC with perineural invasion, as occurred in this patient.^3^

Spontaneous regression is rare but does offer an insight into causes of tumor proliferation and potential therapies.^7^ Many causative mechanisms for spontaneous regression have been proposed, including immunological mechanisms and reduction of immunosuppressive agents.

JAK/signal transducer and activator of transcription is an intracellular pathway that modulates responses to inflammatory cytokines by influencing gene expression. Ruxolitinib is a potent selective JAK 1 and JAK 2 inhibitor. Several mechanisms by which JAK inhibition could induce carcinogenesis have been suggested through its downregulation of immunosurveillance.^4^ Ruxolitinib exposed CD4+ T cells have impaired proliferation, differentiation, and cytokine production. An in vitro study showed recovery of CD4+ T-cell function upon removal of ruxolitinib exposure.^5^ Ruxolitinib also impairs the development, mobility, and cytotoxic activity of dendritic cells.^6^ CD4+ T cells and dendritic cells have antitumor activity.^8^ These immunomodulatory side-effects of ruxolitinib are well recognized and utilized for the treatment of acute graft vs host disease following allogeneic stem cell transplantation.^9^

In a case series of 5 patients who developed Kaposi sarcoma whilst exposed to ruxolitinib, 2 demonstrated spontaneous regression following cessation of ruxolitinib.^10^ We hypothesize that cessation of the immunosuppressive drug ruxolitinib induced the spontaneous regression of this patient's aggressive SCC.

In conclusion, ruxolitinib increases the risk of developing aggressive SCC. Early cessation of ruxolitinib should be considered, particularly for patients with aggressive SCC.

## Conflicts of interest

None disclosed.
